# Access to Procedural Memories After One Year: Evidence for Robust Memory Consolidation in Tourette Syndrome

**DOI:** 10.3389/fnhum.2021.715254

**Published:** 2021-08-12

**Authors:** Eszter Tóth-Fáber, Zsanett Tárnok, Ádám Takács, Karolina Janacsek, Dezső Németh

**Affiliations:** ^1^Doctoral School of Psychology, ELTE Eötvös Loránd University, Budapest, Hungary; ^2^Institute of Psychology, ELTE Eötvös Loránd University, Budapest, Hungary; ^3^Brain, Memory and Language Research Group, Institute of Cognitive Neuroscience and Psychology, Research Centre for Natural Sciences, Budapest, Hungary; ^4^Vadaskert Child and Adolescent Psychiatry Hospital, Budapest, Hungary; ^5^Cognitive Neurophysiology, Department of Child and Adolescent Psychiatry, Faculty of Medicine of the TU Dresden, Dresden, Germany; ^6^Centre of Thinking and Learning, Institute for Lifecourse Development, School of Human Sciences, Faculty of Education, Health and Human Sciences, University of Greenwich, London, United Kingdom; ^7^Lyon Neuroscience Research Center (CRNL), INSERM U1028, CNRS UMR 5292, Université de Lyon, Lyon, France

**Keywords:** Tourette syndrome, memory consolidation, statistical learning, sequence learning, procedural learning

## Abstract

Tourette syndrome is a childhood-onset neurodevelopmental disorder characterized by motor and vocal tics. On the neural level, tics are thought to be related to the disturbances of the cortico-basal ganglia-thalamo-cortical loops, which also play an important role in procedural learning. Several studies have investigated the acquisition of procedural information and the access to established procedural information in TS. Based on these, the notion of procedural hyperfunctioning, i.e., enhanced procedural learning, has been proposed. However, one neglected area is the retention of acquired procedural information, especially following a long-term offline period. Here, we investigated the 5-hour and 1-year consolidation of two aspects of procedural memory, namely serial-order and probability-based information. Nineteen children with TS between the ages of 10 and 15 as well as 19 typically developing gender- and age-matched controls were tested on a visuomotor four-choice reaction time task that enables the simultaneous assessment of the two aspects. They were retested on the same task 5 hours and 1 year later without any practice in the offline periods. Both groups successfully acquired and retained the probability-based information both when tested 5 hours and then 1 year later, with comparable performance between the TS and control groups. Children with TS did not acquire the serial-order information during the learning phase; hence, retention could not be reliably tested. Our study showed evidence for short-term and long-term retention of one aspect of procedural memory, namely probability-based information in TS, whereas learning of serial-order information might be impaired in this disorder.

## Introduction

Tourette syndrome (TS) or Tourette Disorder is a childhood-onset neurodevelopmental disorder characterized by at least one vocal tic and multiple motor tics, which are not explained by medications or other medical conditions (APA, 2013). Tics can be expressed as simple or complex movements or vocalizations that are usually fast, abrupt, and semi-voluntary (APA, 2013). On the neural level, tics are thought to be related to the disturbances of the cortico-basal ganglia-thalamo-cortical (CBGTC) circuits (Peterson et al., 1998, 2003; Stern et al., 2000; Mink, 2001; Albin et al., 2003; Albin and Mink, 2006; Maia and Frank, 2011). On the cognitive level, these circuits are also related to procedural learning (Poldrack and Packard, 2003; Doyon et al., 2009; Janacsek et al., 2020), which is considered to be the basis of skills and habits (Ullman, 2004; Kaufman et al., 2010). It has been proposed that tics and habits have similarities: both are stereotyped actions that are automatically executed and hard to inhibit (Conceição et al., 2017). Several studies have shown enhanced procedural learning, termed procedural hyperfunctioning, in TS (Dye et al., 2016; Takács et al., 2018; Shephard et al., 2019; Tóth-Fáber et al., 2021b). An important question emerges: does procedural hyperfunctioning in TS lead to persistent changes? Processing information does not stop at the end of a learning session, and long-term memory performance is based on the stabilization of encoded information, that is, on the consolidation of information (McGaugh, 2000; Walker, 2005). However, little is known about whether procedural hyperfunctioning persists over the consolidation periods and whether consolidation of procedural information differs in TS and neurotypical controls. In the present study, we focused on this question and investigated the short-term (5-hour) and long-term (1-year) consolidation of procedural information in children with TS.

A potential link has been suggested (Goodman et al., 2014; Takacs et al., 2021) between procedural memory formation and habitual behavior both in everyday life and as a clinical phenomenon. Namely, tics that consist of sequential actions might rely on procedural memory associations. As mentioned above, similar neural networks are involved in the pathophysiology of TS and procedural learning. CBGTC circuits play a key role in the development of tics (Worbe et al., 2010; Goodman et al., 2014) and tics may result from a heightened direct pathway activity relative to the indirect pathway activity in the CBGTC loop (Mink, 2001; Maia and Frank, 2011). Tic-related activation has been shown in the premotor cortex and sensorimotor cortex (Stern et al., 2000; Bohlhalter et al., 2006; Wang et al., 2011), in the supplementary motor area (Bohlhalter et al., 2006; Wang et al., 2011; Worbe et al., 2015), putamen (Stern et al., 2000; Bohlhalter et al., 2006; Wang et al., 2011), globus pallidus (Bohlhalter et al., 2006; Wang et al., 2011) and thalamus (Bohlhalter et al., 2006; Wang et al., 2011; Worbe et al., 2015). Accumulated evidence shows that these brain areas also play a role in procedural learning. Specifically, the formation of skills and habits in procedural memory has been linked to the basal ganglia, particularly to the striatum, and relies on the CBGTC loops (Poldrack and Packard, 2003; Doyon et al., 2009; Janacsek et al., 2020). Given the involvement of similar neural networks in procedural memory and the pathophysiology of TS, alterations of procedural functions can be expected in TS.

Procedural learning enables us to extract the regularities from the environment and underlies the acquisition and storage of skills and habits (Ullman, 2004; Kaufman et al., 2010). Humans are highly proficient in the extraction of transitional probabilities, that is, in the learning of predictive relations between events (i.e., the probability of event B following event A), even when these are non-adjacent (e.g., A – x – B, where the intervening event has no predictive value) (Frost and Monaghan, 2016; Conway, 2020). From a plethora in the environment, different kinds of regularities can be extracted. Two previously proposed regularities in relation to procedural memory are (1) serial order-based information and (2) probability-based, statistical information (Howard et al., 2004; Nemeth et al., 2013). Serial order-based information means that transitional probabilities between the elements are 1.0, which creates a deterministic serial order of events: for instance, event A is always followed by event B. Probability-based information refers to regularities where transitional probabilities are less than 1.0; here, higher transitional probability means higher predictability. Hence, extracting probability-based information enables the differentiation between more and less probable outcomes to learn stochastic relations between events: for instance, when event A is followed by event B in 75% of the cases and followed by event C in 25% of the cases. Although both regularities can be considered as learning of transitional probabilities (also often referred to as statistical learning), prior studies have shown considerable differences between them in healthy young adults (Nemeth et al., 2013; Kóbor et al., 2018; Simor et al., 2019; Takács et al., 2021). They revealed that the learning of serial-order regularities develops rather gradually, whereas the learning of probability-based regularities reaches its plateau in a quick manner (Nemeth et al., 2013; Kóbor et al., 2018; Simor et al., 2019), which is also reflected in the neurophysiological correlates (Kóbor et al., 2018; Takács et al., 2021). In other words, the learning of serial-order regularities occurs relatively slowly, whereas participants acquire probability-based regularities rapidly and then show consistent, stable performance. Prior studies have also shown that successful acquisition of serial-order and probability-based information leads to the formation of long-term memory representations (Romano et al., 2010; Kóbor et al., 2017; Simor et al., 2019; Zavecz et al., 2020; Tóth-Fáber et al., 2021a). Thus, learning of these regularities might influence behavior on a longer timescale and outside of a lab environment as well.

Long-term memory performance is based on consolidation, that is, the stabilization of encoded memory representations (McGaugh, 2000; Walker, 2005). Empirically, consolidation is assessed by the difference in memory performance at the end of a session and at the beginning of the next one, following a delay (i.e., offline period). Consolidation can be revealed by successfully retained knowledge or by delayed gains of performance (i.e., offline learning) after the offline period (Robertson et al., 2004a). Consolidation of any information is a complex process, which can be influenced by the encoded information, time (ultra-fast, short-or long-term consolidation), and the nature of the offline period (i.e., sleep or time spent awake) (Robertson et al., 2004b; Song et al., 2007). Consolidation of serial-order and probability-based regularities has been investigated before, both over short-term (Simor et al., 2019; Zavecz et al., 2020) and long-term offline periods (Romano et al., 2010; Kóbor et al., 2017; Tóth-Fáber et al., 2021a). Romano et al. (2010) and Kóbor et al. (2017) focused on probability-based regularities in neurotypical adults, in both cases after a 1-year offline period. Romano et al. (2010) showed successful retention of probability-based regularities in perceptual-motor skill experts (i.e., videogame and piano players) and non-experts. Kóbor et al. (2017) went beyond the study of Romano et al. (2010) by incorporating interference manipulation into their study design. They have demonstrated that memory representations of probability-based regularities are not only resistant to forgetting over a 1-year offline period but are also resistant to interference. Furthermore, learning of serial-order and probability-based regularities seems to result in long-term memories in the developing mind, as well: Tóth-Fáber et al. (2021a) found evidence for 1-year retention of such regularities in typically developing children and adolescents, thus extended the prior results on adults (Romano et al., 2010; Kóbor et al., 2017) to an age that is crucial in the development of procedural memory (Janacsek et al., 2012; Juhasz et al., 2019; Zwart et al., 2019). In sum, memory representation of these regularities seems to be persistent over a long period of time both in neurotypical adults (Romano et al., 2010; Kóbor et al., 2017) and typically developing children (Tóth-Fáber et al., 2021a). Thus, it is possible to compare procedural memories between typical and atypical development after a long offline period. Crucially, extending the testing time to 1 year allows us to close the bridge between the time scale of lab experiments (typically hours or days) and real-world observations (i.e., when learning a new skill or developing a habit).

Several studies focused on procedural learning in TS with most showing intact (Channon et al., 2003; Takács et al., 2017) or even enhanced procedural functions (Walenski et al., 2007; Dye et al., 2016; Takács et al., 2018; Shephard et al., 2019; Tóth-Fáber et al., 2021b). Takács et al. (2018), Shephard et al. (2019), and Tóth-Fáber et al. (2021b) all employed variations of a well-known procedural learning task, the serial reaction time task (SRTT). Consequently, Shephard et al. (2019) showed enhanced learning of deterministic serial-order information, whereas Takács et al. (2018) and Tóth-Fáber et al. (2021b) showed enhanced learning of probability-based information in children and adolescents with TS. In conjunction with the online learning tasks, procedural hyperfunctioning has also been shown in tasks that measure the access to previously established procedural information, such as grammatical rules or vocabulary (Ullman, 2004; Walenski et al., 2007). For example, in the study of Walenski et al. (2007), compared to typically developing controls, children with TS showed faster production of rule-governed past tenses and faster naming of manipulated objects—both of which have been linked to procedural memory (Ullman, 2004). Additionally, Dye et al. (2016) provided evidence for enhanced access to established information in the phonological domain of language. They used a non-word repetition task that involved rule-governed grammatical (de)composition of non-words; therefore, it relied, at least in part, on procedural memory. Children with TS showed faster repetition of non-words than typically developing controls on this task. These findings suggest that not only procedural learning but also access to previously consolidated procedural knowledge may be enhanced in TS. This raises the question of whether the consolidation of procedural information is also atypical in this disorder.

Consolidation of procedural information in TS has not received much attention in previous research. Takács et al. (2018) incorporated a 16-hour offline period in their study design and investigated the learning and consolidation of probability-based regularities in children with TS. The TS group showed superior learning, but they showed greater forgetting following the overnight offline period than the typically developing controls. When controlling for the learning differences and comparing overnight changes as a function of prior knowledge, the TS and control groups showed comparable performance. Nevertheless, the differences in learning between the TS and typically developing groups make the interpretation difficult, and, as Takács et al. (2018) suggested, these results should be handled as inconclusive. According to our knowledge, there are no other studies up to date that directly investigated procedural consolidation in TS. In sum, it remains unresolved whether atypical procedural learning in TS leads to altered consolidation of procedural memories.

The present study focuses on the short- (5-hour) and long-term (1-year) consolidation of two aspects of procedural memory, namely serial-order and probability-based information in children with TS. To test this, we employed a widely used procedural learning task, namely the cued version of the Alternating Serial Reaction Time (ASRT) task, which enables us to measure the acquisition and consolidation of the two regularities simultaneously (Nemeth et al., 2013). Children with TS and age- and gender-matched typically developing controls performed the cued ASRT task in three sessions. To investigate the short-term consolidation of serial-order and probability-based information, the first two sessions took place on the same day with a 5-hours offline period between them. To test the 1-year consolidation of the two regularities, the third session was administered following a 1-year offline period. Hence, this explorative study aims to examine both the short-term and the long-term consolidation processes in children with TS.

## Materials and Methods

### Participants

Twenty children diagnosed with TS between the ages of 10 and 15 participated in our study. They were recruited through a child and adolescent psychiatry hospital in Budapest, Hungary. They had been diagnosed with TS based on the DSM-V criteria (APA, 2013). Diagnoses were made by a team of child psychiatrist, clinical psychologist and special education teacher after a one-week-long observation in the hospital. One participant had to be excluded from the analyses as they consistently showed extremely low average accuracy on the regularity extraction task (more than 3 times the interquartile range from the quartiles; Tukey, 1977). Therefore, the final TS sample consisted of 19 children (16 boys and three girls). Demographic and clinical data of the TS participants are reported in Table 1. Three children had comorbid attention deficit hyperactivity disorder (ADHD) and one child had comorbid ADHD and obsessive-compulsive disorder (OCD). We did not exclude these participants from the analyses as ADHD and OCD are highly common in TS (Robertson et al., 2017). Participants did not have any other psychiatric or neurodevelopmental disorders. Three children were taking medication during either time of testing: one child was taking atomoxetine during the first testing, and two children were taking atomoxetine during the second testing. A subgroup of the TS children had been examined in the study of Tóth-Fáber et al. (2021b) (the overlap between the two samples is 81%), however, a new control group had been recruited due to difficulties in assessing the original control group 1 year later.

**TABLE 1:** Demographic and clinical data of the participants.

	**Group**			
	**TS (*n* = 19)**		**TD (*n* = 19)**	
	***M***	***SD***	***M***	***SD***
Age on the first testing day	11.95 years	1.27 years	11.79 years	1.48 years
School grade on the first testing day	5.68	1.29	5.95	1.47
Caregivers’ average formal education	16.24 years	2.85 years	16.45 years	3.08 years
YGTSS total score on the first testing day	18.21	8.61	–	–
YGTSS total score on the second testing day	17.58	9.35	–	–

*YGTSS = Yale Global Tic Severity Scale.*

Seventy-eight typically developing (TD) children were recruited from local schools [note that the analyses on this sample had been reported in Tóth-Fáber et al. (2021a)]. From this group, we matched 19 children one-to-one to the TS participants based on age and gender (16 boys and three girls). The pairs had an age gap maximum of 6 months and were in the same school grade. None of the matched controls had any psychiatric, neurological, or neurodevelopmental disorders based on parental reports. All participants had normal or corrected-to-normal vision. Demographic data of the TD participants are reported in Table 1.

Caregivers of all participants completed a parental questionnaire regarding socioeconomic status (SES). SES was determined by the number of years the caregivers spent in formal education and it is reported in Table 1. Caregivers’ average formal education was calculated based on both parents’ education. In case of one participant in the TS group and three participants in the TD group, we only had information about one caregiver. In the TS group, data of two participants are missing.

Caregivers of all participants provided informed written consent, and children assented to participate in the study before enrollment. The study was approved by the research ethics committee of Eötvös Loránd University, Budapest, Hungary, and was conducted in accordance with the Declaration of Helsinki.

### Tasks

#### Alternating Serial Reaction Time (ASRT) Task

**FIGURE 1:**
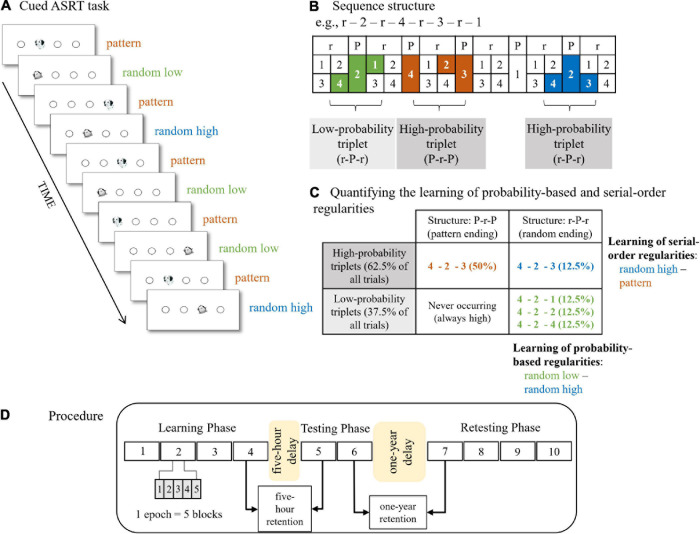
The cued Alternating Serial Reaction Time (ASRT) task and experimental procedure. **(A)** Pattern and random trials were presented in an alternating fashion. Pattern trials were represented by a dog’s head and random trials were represented by a penguin. **(B)** An example of the sequence structure. In the example sequence, numbers mark pattern trials and “r” marks a randomly selected location out of the four possible locations. The alternating presentation of trials makes some runs of three consecutive trials (called triplets) more probable than others, labeled as high-probability and low-probability triplets, respectively. High-probability triplets can end either with a pattern or a random trial, whereas low-probability triplets always end with a random trial. Therefore, we can differentiate pattern triplets which are always of high probability (orange shading in **panel B** and orange font in **panel C**), random high-probability triplets (blue shading in **panel B** and blue font in **panel C**), and random low-probability triplets (green shading in **panel B** and green font in **panel C**). **(C)** Quantifying the underlying learning processes in the task. Learning of probability-based regularities is quantified by contrasting the RTs on the random high and random low trials (blue vs. green, the right column of the table). Learning of serial-order regularities is calculated by contrasting the RTs on the pattern and random high trials (orange vs. blue, the top row of the table). **(D)** The design of the experiment. The experiment consisted of three sessions. The Learning Phase and Testing Phase were administered on the same day with a 5-hour offline period between them. The Learning Phase was composed of four epochs (one epoch contained 5 blocks, and each block consisted of 85 stimuli) and the Testing Phase was composed of two epochs. The four-epoch-long Retesting Phase was administered ca. 1 year later. Figures 1A–C are adapted from Nemeth et al. (2013) and Zavecz et al. (2020), Figure 1D is adapted from Kóbor et al. (2017).

The cued version of the Alternating Serial Reaction Time (ASRT) task (Howard and Howard, 1997; Nemeth et al., 2013) was employed to measure the extraction of probability-based and serial-order regularities. The ASRT task has adequate test-retest reliability on neurotypical adult population (Stark-Inbar et al., 2016). In this task, participants see four equally spaced empty circles which are horizontally arranged. A stimulus (either a dog’s head or a penguin) occurs in one of the empty circles (Figure 1A). Participants were instructed to press the corresponding key (Z, C, B, or M) on a QWERTY keyboard as accurately and as fast as they could. The response-to-stimulus interval was set to 120 ms.

In the task, pattern and random stimuli appeared in an alternating fashion. Pattern stimuli appeared following a predetermined sequence, whereas random stimuli could appear in one of the possible locations (i.e., empty circles). The stimuli were presented in blocks with 85 trials in each block. A block started with five random trials for practice, followed by an eight-element alternating sequence presented ten times. The alternating sequence consisted of pattern and random trials (e.g., 1-r-2-r-4-r-3-r, where numbers indicate one of the four circles on the screen and “r” indicates a randomly selected circle out of the four possible ones). In the cued ASRT task, participants are informed about the presence of the sequence, and their attention is drawn to the alternating sequence by marking the pattern and random trials with different visual stimuli. In our study, a picture of a dog denotes pattern trials, and a picture of a penguin represents random trials. Participants were not informed about the exact sequence, but they were instructed to find the pattern defined by the dogs’ appearance to improve their performance. For each participant, one of the six different sequence permutations was selected in a pseudo-random fashion, and the presence of the permutations was counterbalanced across participants and groups. For a given participant, the sequence permutation was the same across the epochs and the sessions. Note that the permutations can start at any location (e.g., 1-r-2-r-3-r-4-r and 2-r-3-r-4-r-1-r are identical sequence permutations).

Due to the alternating sequence (i.e., pattern and random elements occurring in an alternating fashion), some runs of three consecutive trials (referred to as triplets) were more probable than others. For example, if the sequence is 1-r-2-r-4-r-3-r, triplets such as 1-X-2, 2-X-4, 4-X-3, 3-X-1 (where X represents the middle element of the triplet) occur with a higher probability as their first and third elements could have been either pattern or random. This means that for example 4-X-3 can appear both as 4-2-3 (pattern – random – pattern) where the first and last elements are part of the predetermined sequence and as 4-2-3 (random – pattern – random) where the first and last elements are random, and the middle element is part of the predetermined sequence. However, triplets such as 3-X-2 or 4-X-2 were less probable as their first and third elements could have been only random (that is, random – pattern – random structure). More probable triplet types are referred to as “high-probability” triplets, while the less probable ones are labeled as “low-probability” triplets (Howard and Howard, 1997; Nemeth et al., 2013). Each trial was categorized as the last element of either a high-probability or a low-probability triplet in a sliding window manner (i.e., one trial was the last element of a triplet, but it was also the middle and the first element of the two consecutive triplets, respectively). Another crucial feature of the trials is whether they belong to pattern or random elements (i.e., marked by a picture of a dog or a penguin). There are 64 unique triplets in the task, including all pattern-ending (50%) and random ending (50%) triplets. Sixteen of these unique triplets are high-probability and 48 triplets are low-probability. As high-probability triplets can occur as pattern-ending triplets (50% of all trials) and by 1/4 chance as random-ending triplets (12.5% of all trials), these triplets constitute 62.5% of all trials (Figure 1C). Low-probability triplets constitute 37.5% of all trials. On the level of unique triplets, high-probability triplets are five times more probable than low-probability triplets (4% [62.5%/16] vs. 0.8% [37.5%/48]). In sum, three trial types could be differentiated: (1) trials that belonged to the predefined sequence and appeared as the last element of a high-probability triplet called *pattern trials*; (2) random elements that belong to a high-probability triplet labeled as *random high trials*; and (3) random elements that are the last element of a low-probability triplet called *random low trials* (Figures 1B,C).

In the cued ASRT task, the acquisition of probability-based and serial-order regularities, also referred to as statistical and sequence learning, respectively, can be measured simultaneously (Nemeth et al., 2013). Learning of probability-based regularities was measured by the difference in reaction times (RTs) between random high and random low trials, greater learning was defined by faster RTs on random high than on random low trials. As both trials were random, they shared the same sequence properties but differed in statistical properties as they corresponded to the last element of a high-probability or a low-probability triplet, respectively. Learning of serial-order regularities was quantified by the difference in RTs between pattern and random high trials, greater learning was determined by faster RTs on pattern than on random high trials. The two trial types shared the statistical properties as they both were the last element of a high-probability triplet, however, they differed in sequence properties as pattern trials belonged to the predefined sequence (Figure 1C).

At the beginning of the ASRT task, participants were instructed to discover the pattern of the dogs’ appearance. At the end of each block, awareness of the serial-order structure was assessed. Participants were asked to type the order of the dogs using the corresponding keys. The post-block sequence report lasted until 12 consecutive responses, which ideally was the 4-element sequence three times. The post-block sequence reports after the last five blocks of the Learning Phase (see Procedure) were used to measure awareness of the sequence. We calculated how many out of the 12 consecutive responses were correct after each block; hence, we created a percentile variable. The mean of these five percentile variables was calculated for each participant, and we termed this variable as *explicit knowledge* of the sequence structure.

#### Yale Global Tic Severity Scale (YGTSS)

Tic severity was assessed by a widely used and conventional measurement, namely the Yale Global Tic Severity Scale (Leckman et al., 1989). YGTSS is a semi-structured interview, which rates the number, frequency, complexity, intensity, and interference of motor and vocal tics separately on a scale of zero to five. The Total Score reported here contains the motor and vocal tic scores with a maximum score of 50. Tic severity was assessed two times: on the first testing day and 1 year later. Tic severity scores represent values from the week prior to the experiment.

### Procedure

The study consisted of three sessions (Figure 1D). The first two sessions took place on the same day with a 5-hour-long offline period between them. Children completed the learning session at the beginning of a school day and returned after their lunch break (that is, 5 hours later). The third session was administered ca. 1 year later (*M*_delay_ = 53.78 weeks, *SD*_delay_ = 3.11 weeks, between 47.95 and 60.57 weeks). Participants were assessed on the ASRT task in all three sessions. The ASRT task was presented in blocks. During the statistical analyses, blocks were collapsed into epochs, with each epoch containing five blocks. The Learning Phase consisted of 20 blocks (i.e., four epochs), the Testing Phase was composed of 10 blocks (i.e., two epochs) and the Retesting Phase again contained 20 blocks (i.e., four epochs). After the first testing day, participants were not informed that the ASRT task would be administered again 1 year later.

### Statistical Analyses

Statistical analyses were carried out by SPSS version 25.0 software and by JASP 0.9.2.0. software. We followed protocols outlined in previous studies (e.g., Nemeth et al., 2013; Kóbor et al., 2018; Simor et al., 2019). First, we collapsed the blocks into epochs of five blocks to facilitate data processing. The first epoch contained blocks 1–5, the second epoch contained blocks 6–10, and so forth. Hence, the Learning Phase consisted of four epochs, the Testing Phase consisted of two epochs and the Retesting Phase consisted of four epochs. Epochs are referred to consecutively (from 1 to 10).

Each trial was defined as the last element of a pattern, random high or random low triplet (Howard and Howard, 1997; Nemeth et al., 2013; also see the task’s description above). We calculated the median RT for correct responses separately for the three trial types, for each participant and each epoch. Based on prior studies, we excluded two types of low-probability triplets: repetitions of a single element (e.g., 111, 222) and trills (i.e., triplet starting with and ending with the same element, e.g., 121, 242) as individuals often show pre-existing tendencies toward them (Howard et al., 2004).

Based on the three trial types, learning of probability-based and serial-order regularities can be quantified (Nemeth et al., 2013). Learning of probability-based regularities was defined as the difference in RT between random high and random low trials (RT for random low trials minus RT for random high trials). Learning of serial-order regularities was quantified as the RT difference between pattern and random high trials (RT for random high trials minus RT for pattern trials). Hence, higher scores indicate better learning/memory of probability-based or serial-order information. In conjunction with the learning and memory scores, we also calculated an offline change score separately for knowledge of probability-based and serial-order regularities. The short-term offline change score was calculated by subtracting the memory score in Epoch 4 from the memory score in Epoch 5, therefore, it shows the change over the 5-hour offline period. The long-term offline change score was calculated by subtracting the memory score in Epoch 6 from the memory score in Epoch 7, therefore, it shows the change over the 1-year offline period. In both cases, negative scores show forgetting and positive scores indicate offline learning. To assess learning and the retention of knowledge, repeated-measures ANOVAs and paired-samples *t*-tests were conducted on RT data, separately for probability-based and serial-order based regularities. The Greenhouse-Geisser epsilon (ε) correction was used when necessary. Original *df* values and corrected *p* values (if applicable) are reported with partial eta-squared (η*^2^_*p*_*) as a measure of effect size. For correlation analyses, in case of normal distribution, Pearson’s correlation was employed. When the assumption of normal distribution was violated, Spearman correlation was used for frequentist statistics and Kendall’s Tau-b correlation was used for Bayesian statistics.

Concurrently with the frequentist analyses, Bayesian paired-samples *t*-tests and independent-samples *t*-tests were performed, and Bayes Factors (BF) were calculated for the relevant comparisons. The *BF* is an appropriate tool to conclude whether the data support the null (H_0_) or alternative (H_1_) hypothesis (Wagenmakers et al., 2011). *BF*s can be particularly relevant in memory consolidation studies where retention is indicated by evidence supporting the H_0_ rather than H_1_ (Dienes, 2014), as H_0_ means that the memory scores before and after the offline period are similar and H_1_ means that the memory scores differ. Here, we report *BF*_01_ values. According to Wagenmakers et al. (2011), *BF*_01_ values between 1 and 3 suggest anecdotal evidence, values between 3 and 10 indicate substantial evidence and values larger than 10 mean strong evidence for H_0_. Values between 1 and 1/3 indicate anecdotal evidence, values between 1/3 and 1/10 indicate substantial evidence, and values below 1/10 suggest strong evidence for H_1_. Values around 1 do not support either hypothesis.

## Results

### Prerequisite of Memory Consolidation

Significant learning preceding the offline period is a prerequisite of assessing memory consolidation (Robertson, 2009; Kóbor et al., 2017). Thus, we conducted mixed-design ANOVAs on the Learning Phase to test whether significant learning of probability-based and/or serial-order regularities occurred in both the TS and TD groups. ANOVAs were conducted on RT data, separately for the two types of regularities.

**FIGURE 2:**
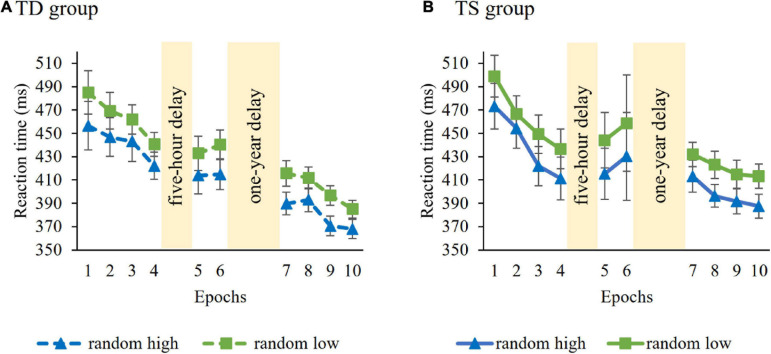
Temporal dynamics of learning of probability-based regularities across epochs and sessions in the **(A)** TD group and **(B)** TS group. Dashed lines represent the TD group, continuous lines represent the TS group. RT values as a function of the epoch (1–10) and trial types (random high vs. random low) are presented. Blue lines with triangle symbols indicate RTs on the random high trials, green lines with square symbols indicate RTs on the random low trials. Learning is quantified by the gap between blue and green lines; the greater gap between the lines represents better learning. Error bars denote standard error of mean.

**Learning of probability-based regularities** in the Learning Phase were tested with a mixed-design ANOVA on RT with GROUP (TS vs. TD) as a between-subjects factor and PROBABILITY (random high vs. random low) and EPOCH (1–4) as within-subject factors. Average RTs (i.e., irrespective of trial types) were similar in the control and TS groups [main effect of GROUP, *F*(1, 36) = 0.006, *p* = 0.94). RTs gradually decreased as the task progressed, irrespective of trial types [main effect of EPOCH, *F*(3, 108) = 20.62, *p* < 0.001, η*^2^_*p*_* = 0.36]. The ANOVA revealed significant learning of probability-based information [main effect of PROBABILITY, *F*(1, 36) = 83.48, *p* < 0.001, η*^2^_*p*_* = 0.70], participants showed faster responses to random high (*M* = 441.20 ms) than to random low trials (*M* = 463.52 ms). The TS and TD groups did not differ from each other either in overall learning [GROUP × PROBABILITY interaction, *F*(1, 36) = 0.02, *p* = 0.90; Figure 2] or in the trajectory of learning [GROUP × PROBABILITY × EPOCH interaction, *F*(3, 108) = 1.06, *p* = 0.36]. Other interactions were also not significant (all *p*s > 0.13). Successful learning in both groups ensure that the analyses of short-term and long-term consolidation of probability-based regularities across groups are justified.

**FIGURE 3:**
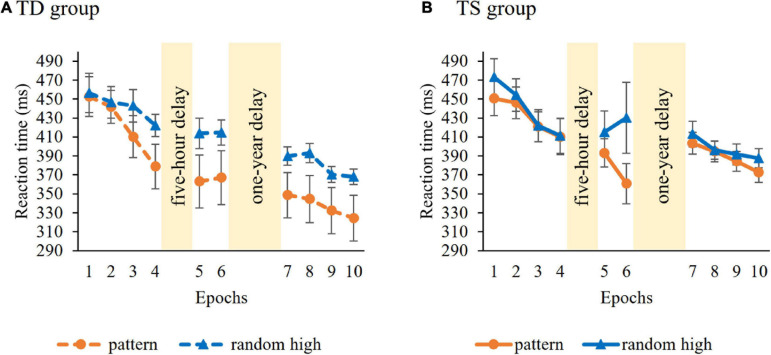
Temporal dynamics of learning of serial-order regularities across epochs and sessions in the **(A)** TD group and **(B)** TS group. Dashed lines represent the TD group, continuous lines represent the TS group. RT values as a function of the epoch (1–10) and trial types (pattern vs. random high) are presented. Orange lines with circle symbols indicate RTs on the pattern trials, blue lines with triangle symbols indicate RTs on the random high trials. Learning is quantified by the gap between orange and blue lines; the greater gap between the lines represents better learning. Error bars denote standard error of mean.

**Learning of serial-order regularities** during the Learning Phase was tested similarly, with a mixed-design ANOVA on RT with GROUP (TS vs. TD) as a between-subjects factor and ORDER (pattern vs random high) and EPOCH (1–4) as within-subject factors. Average RTs (i.e., irrespective of trials types) did not differ in the control and TS groups [main effect of GROUP, *F*(1, 36) = 0.04, *p* = 0.85]. RTs gradually decreased as the task progressed, irrespective of trial types [main effect of EPOCH, *F*(3, 108) = 28.55, *p* < 0.001, η*^2^_*p*_* = 0.44]. The ANOVA showed overall significant learning [main effect of ORDER, *F*(1, 36) = 6.59, *p* = 0.015, η*^2^_*p*_* = 0.16], participants showed faster RTs to pattern (*M* = 426.47 ms) compared to random high trials (*M* = 441.20 ms). Importantly, however, the groups differed in the trajectory of learning [indicated by the GROUP × ORDER × EPOCH interaction, *F*(1, 36) = 5.03, *p* = 0.01, η*^2^_*p*_* = 0.12, Figure 3]. Other interactions were not significant (all *p*s > 0.27). To further examine the three-way interaction, we investigated the learning of serial-order regularities separately in the TS and TD groups. Hence, we conducted an ANOVA on RT with ORDER (pattern vs. random high) and EPOCH (1–4) separately for the two groups. In the TS group, the ANOVA did not reveal learning [non-significant main effect of ORDER, *F*(1, 18) = 3.58, *p* = 0.08; non-significant ORDER × EPOCH interaction, *F*(3, 54) = 2.25, *p* = 0.09]. The TD group did not show significant learning either [non-significant main effect of ORDER, *F*(1, 18) = 3.99, *p* = 0.06; non-significant ORDER × EPOCH interaction, *F*(3, 54) = 3.35, *p* = 0.07]. Importantly, these results suggest that the groups did not successfully acquire the serial-order information during the Learning Phase, therefore, the prerequisite of assessing memory consolidation was not fulfilled. The lack of significant learning calls into question the applicability of retention analyses concerning serial-order regularities. Hence, from this point on, we focus on consolidation of probability-based information and report the analysis on the consolidation of serial-order regularities in the Supplementary Material.

Regarding serial-order learning, we also tested the explicit knowledge of the sequence measured by the post-block sequence reports and whether it is different in the TS and control groups. Due to the violation of normal distribution, non-parametric Mann-Whitney *U* test was used to contrast explicit knowledge in the TS and control groups. The two groups showed similar explicit knowledge (*U* = 151.5, *z* = −0.92, *p* = 0.36; *M*_control_ = 79.23%, *M*_TS_ = 88.42%).

### Short-Term (Five-Hour) Consolidation of Knowledge of Probability-Based Regularities

To examine the 5-hour consolidation of knowledge of probability-based regularities, we conducted a mixed-design ANOVA on RT with GROUP (TS vs. TD) as between-subjects factor and PROBABILITY (random high vs. random low) and EPOCH (4 vs. 5) as within-subject factors.

**FIGURE 4:**
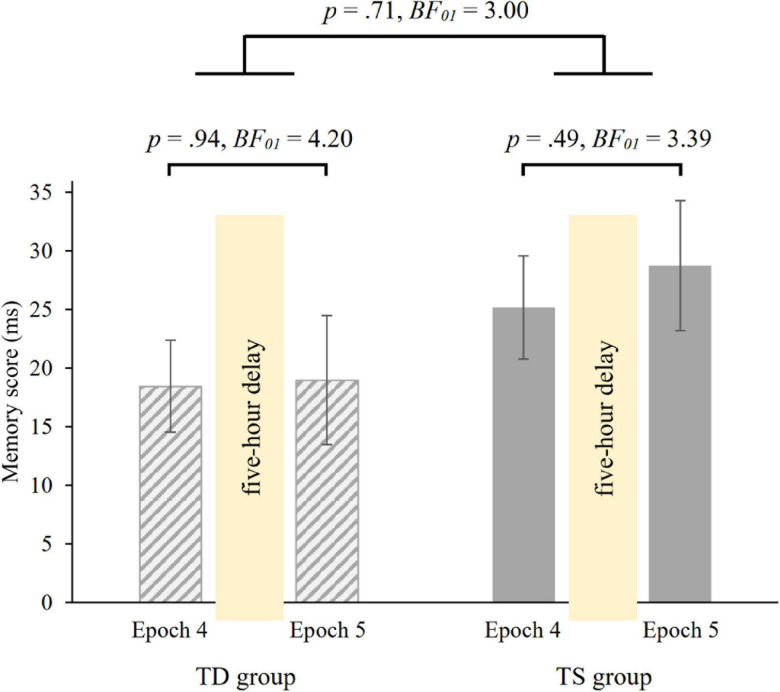
5-hour retention of knowledge of probability-based regularities in the TD and TS groups. Memory scores were measured by RT values for the last epoch of the Learning Phase (Epoch 4) and the first epoch of the Testing Phase (Epoch 5). Error bars denote the standard error of mean.

Overall, irrespective of epochs and group, participants were faster on random high (*M* = 415.70 ms) than on random low trials (*M* = 438.53 ms) [main effect of PROBABILITY, *F*(1, 36) = 66.37, *p* < 0.001, η*^2^_*p*_* = 0.65]. The ANOVA revealed, that over groups, the memory scores did not change in the 5-hour offline period [non-significant PROBABILITY × EPOCH interaction, *F*(1, 36) = 0.25, *p* = 0.62, *BF*_01_ = 5.08], with similar memory scores in the 4th (*M* = 21.80 ms) and in the 5th (*M* = 23.86 ms) epochs. Importantly, the groups did not differ in retention [non-significant GROUP × PROBABILITY × EPOCH interaction, *F*(1, 36) = 0.14, *p* = 0.71, Figure 4; Bayesian independent samples *t*-tests conducted on the short-term offline change score *BF*_01_ = 3.004, short-term offline change scores: *M*_TS_ = 3.58 ms, *M*_TD_ = 0.53 ms]. Other main effects or interactions were also not significant (all *p*s > 0.15). Furthermore, we compared the memory scores in Epoch 4 and Epoch 5 separately in the two groups with paired-samples *t*-tests. Both groups showed retention of probability-based regularities [TD group: *t*(18) = −0.08, *p* = 0.94, *BF*_01_ = 4.20, *d* = −0.02; TS group: *t*(18) = −0.70, *p* = 0.49, *BF*_01_ = 3.39, *d* = −0.16, see also Figure 4].

### Long-Term (One-Year) Consolidation of Knowledge of Probability-Based Regularities

**FIGURE 5:**
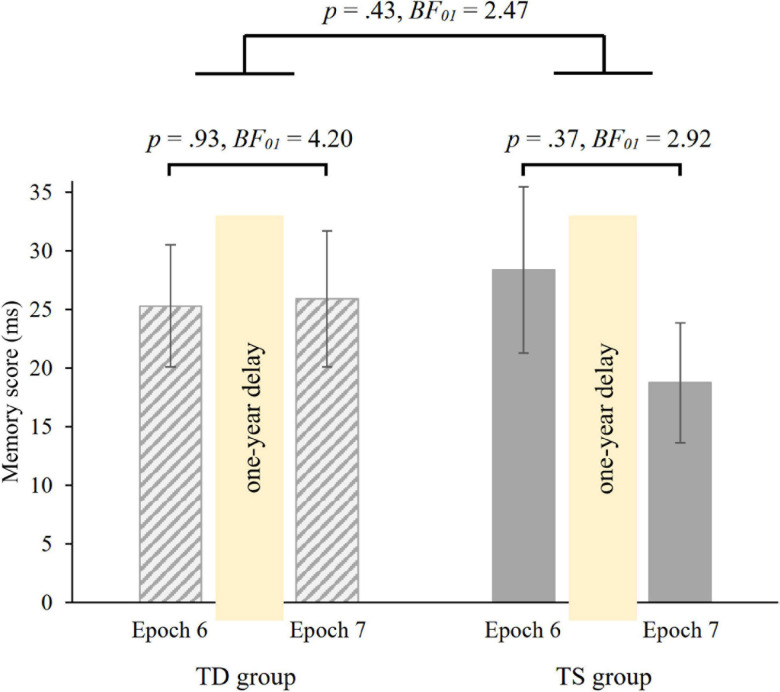
1-year retention of knowledge of probability-based regularities in the TD and TS groups. Memory scores were measured by RT values for the last epoch of the Testing Phase (Epoch 6) and the first epoch of the Retesting Phase (Epoch 7). Error bars denote the standard error of mean.

To investigate the 1-year consolidation of knowledge of probability-based regularities, we run a mixed-design ANOVA on RT with GROUP (TS vs. TD) as between-subjects factor and PROBABILITY (random high vs. random low) and EPOCH (6 vs. 7) as within-subject factors. Overall, irrespective of epochs and group, participants showed faster RTs on random high (*M* = 412.08 ms) than on random low trials (*M* = 436.68 ms) [main effect of PROBABILITY, *F*(1, 36) = 87.75, *p* < 0.001, η*^2^_*p*_* = 0.71]. The ANOVA revealed retained memory of probability-based regularities after the 1-year delay [non-significant PROBABILITY × EPOCH interaction, *F*(1, 36) = 0.496, *p* = 0.49, *BF*_01_ = 4.53], memory scores were similar in the 6th (*M* = 26.85 ms) and in the 7th (*M* = 22.34 ms) epochs. Importantly, memory scores were similar in the TS and TD groups [non-significant GROUP × PROBABILITY × EPOCH interaction, *F*(1, 36) = 0.64, *p* = 0.43, Figure 5; Bayesian independent samples *t*-tests conducted on the long-term offline change score *BF*_01_ = 2.47, long-term offline change scores: *M*_TS_ = −9.63 ms, *M*_TD_ = 0.61 ms]. Other main effects and interactions were also not significant (all *p*s > 0.20). Furthermore, we compared the memory scores in Epoch 6 and Epoch 7 separately in the two groups with paired-samples *t*-tests. Both groups showed retention of probability-based regularities [TD group: *t*(18) = −0.08, *p* = 0.93, *BF_01_* = 4.20, *d* = −0.02; TS group: *t*(18) = 0.91, *p* = 0.37, *BF*_01_ = 2.92, *d* = 0.21, see also Figure 5].

### The Relationship Between Tic Severity and Consolidation of Knowledge of Probability-Based Regularities

In the TS group, we measured the severity of present tics on the first testing day as well as 1 year later, on the second testing day. This way, we could assess the change in tic severity over the 1-year offline period. We subtracted the total score of tic severity on the second testing day (i.e., after the 1-year offline period) from the total score of tic severity on the first testing day. Therefore, positive scores mean positive change over the 1-year offline period, and negative scores mean that tics became more severe. The mean total scores on the first and second testing days are reported in Table 1. The mean of the change in tic severity was 0.63 (*SD* = 9.55).

To evaluate the relationship between tic severity and consolidation of knowledge of probability-based regularities, we correlated short- and long-term offline change scores and tic severity on the first testing day. Severity of the present tics on the first testing day did not correlate either with short-term offline change score [*rs*(19) = −0.10, *p* = 0.68, *BF*_01_ = 3.22], or with long-term offline change score [*rs*(19) = −0.07, *p* = 0.77, *BF*_01_ = 3.32]. Moreover, we correlated the long-term offline change score of knowledge of probability-based regularities with the change in tic severity over the 1-year offline period and found no correlation between the variables [*rs*(19) = −0.19, *p* = 0.43, *BF*_01_ = 2.57].

## Discussion

The present study aimed to investigate the short-term (5-hour) and long-term (1-year) consolidation of two aspects of procedural memory, namely probability-based and serial-order regularities, in children with Tourette syndrome and neurotypical peers. We employed the cued ASRT task, which measures the two aspects simultaneously. We have shown retained knowledge of probability-based information: participants acquired the probability-based regularities, then successfully retained them both after the 5-hour and 1-year offline period. Children with TS and matched typically developing controls showed comparable retention of knowledge of probability-based regularities. These results were supported by Bayesian statistics as well, strengthening the evidence for successful 5-hour and 1-year retention in both groups. Concerning serial-order regularities, the prerequisite of assessing memory consolidation was not fulfilled as the groups did not acquire the serial-order information. Hence, consolidation of serial-order information could not be reliably tested here. Nevertheless, we presented these results in the Supplementary Materials showing successful retention in both groups.

Previous studies already demonstrated retained memory representation of probability-based information in neurotypical adults following a 1-year offline period using the ASRT task (Romano et al., 2010; Kóbor et al., 2017). Importantly, evidence for successful retention was presented in neurotypical children as well in the study of Tóth-Fáber et al. (2021a). Altogether, these studies suggest that 1-year consolidation of probability-based regularities seems to be comparable between children and adults, supporting the age invariance model in the consolidation of such regularities. Our results corroborate these prior findings and also demonstrates intact 1-year consolidation of probability-based information in children with TS, suggesting that procedural memory is robust in this neurodevelopmental disorder.

The intact consolidation of knowledge of probability-based regularities in TS is in line with the results of prior studies. Takács et al. (2018) employed the uncued version of the ASRT task and probed learning and consolidation of probability-based regularities in TS. Children with TS showed superior learning, however, following a 16-hour offline period, they showed greater forgetting than their neurotypical peers. Importantly, group differences in learning itself can lead to group differences in consolidation. When controlling for learning differences, the TS and control group showed similar changes in knowledge of probability-based regularities overnight, suggesting that consolidation is comparable between the groups. The present study replicates and goes beyond the results of Takács et al. (2018): (1) our results also showed comparable short-term (5-hour) consolidation, and (2) we showed intact 1-year retention of knowledge of probability-based regularities in TS.

Consolidation of procedural memory representations in TS has also been indirectly examined with language-based tasks that measure the access to previously established procedural information. Walenski et al. (2007) showed faster production of rule-governed past tenses and faster naming of manipulated objects in TS, whereas production of irregular past tenses and naming non-manipulated objects were similar between the TS and typically developing groups. The former processes rely on procedural memory and the latter processes are related to declarative memory (Ullman, 2004). In conjunction with these results, Dye et al. (2016) found evidence for enhanced access to established procedural information in TS in a non-word repetition task: children with TS could repeat non-words in a faster manner than typically developing controls. In sum, the results of both Walenski et al. (2007) and Dye et al. (2016) suggested enhanced access to previously consolidated procedural information in TS, whereas our study showed intact procedural memory consolidation. This discrepancy might be explained by the differences between the employed tasks. Although processing of probability-based regularities is important in language (e.g., Saffran et al., 1996; Thompson and Newport, 2007; Misyak et al., 2010), the ASRT task employed in the current study and the language-based tasks used in the prior studies (Walenski et al., 2007; Dye et al., 2016) show some differences. Both language-based studies used stimuli or measured processes participants have a prior knowledge of: Walenski et al. (2007) used words as stimuli and Dye et al. (2016) measured rule-governed (de)composition of words. In contrast, participants had no prior knowledge of the stimuli and the underlying structure presented in the ASRT task. Moreover, participants have a repeated exposure to the stimuli in the language-based tasks besides the experimental sessions, hence, the stimuli are retained, and therefore the memory representations are reinforced regularly, whereas participants met with the ASRT task solely in the experimental sessions. Speculatively, it is possible that regular practice in the offline period is needed for the enhancement seen in the language-based studies (Walenski et al., 2007; Dye et al., 2016). Further studies are warranted to test this possibility by investigating language-related processes and extraction of regularities during the lifespan of TS patients.

Long-term stability of procedural memories has potential clinical and educational implications. Procedural memory underlies the acquisition of cognitive, motor and social skills, such as language learning or sports (Kaufman et al., 2010; Frith and Frith, 2012) and is related to habits as well (Goodman et al., 2014; Takacs et al., 2021). Importantly, a 1-year long offline period that has been used in the current study can resemble real-word observations. Namely, learning a new skill or developing a habit happens over a longer stretch of time than a timescale of a lab visit. Our results suggest that children with TS have stable memory representations of procedural knowledge without additional practice during a long time interval and their performance is comparable with TD children. These robust memory representations of procedural knowledge could manifest in everyday settings in the following way: children with TS might be better in learning a new skill (as suggested by prior studies on procedural learning, Takács et al., 2018; Tóth-Fáber et al., 2021b) and they can be also successful in maintaining and remembering those skills, as the current study suggests. As for clinical settings, behavioral therapies are first-line treatments for reducing tics. This method can potentially benefit from a dovetailed knowledge of how stable the acquired skills are in TS. For instance, in habit reversal training (Piacentini and Chang, 2005), when feeling the urge to tic, patients learn to perform an adequate, antagonist action that is physically incompatible with the tic. Over time and practice, when feeling the urge, patients will carry out the adequate action instead of the tic, hence, the urge – tic association will be replaced by the urge – adequate action association. It is conceivable that the new association results in a stable memory representation just as tics and procedural knowledge. Relatedly, Petruo et al. (2020) examined the effect of behavioral therapy on procedural associations and inhibitory control in adolescents with TS and typically developing peers. They employed the Comprehensive Behavioral Intervention for Tics (CBIT, Piacentini et al., 2010), which is a complex behavioral therapy consisting of psychoeducation, relaxation and habit reversal training (HRT). Procedural associations and inhibitory control were tested preceding and following the intervention. Participants with TS showed worse inhibitory control than TD peers during the pretest, however, performance was comparable between the groups during the posttest. These results suggest that CBIT reduced the rigid procedural associations in TS, resulting in successful inhibitory control. The authors concluded that, in conjunction with reducing tics, CBIT/HRT might normalize alterations in higher level cognitive functions (Petruo et al., 2020). Furthermore, one intriguing but so far neglected area which has great clinical implications is the rewiring of memory representations (Szegedi-Hallgató et al., 2017) in TS. Previous studies have suggested that memory representations of procedural knowledge might be overstable in TS (Shephard et al., 2019; Takacs et al., 2021), which would result in higher resistance to interference. This would lead to more difficulties in rewiring/overwriting established associations. Future studies should test how well patients with TS can overwrite associations and how it relates to behavioral therapies. In sum, it is important to note that the abovementioned clinical implications are tentative and future studies are necessary to investigate the relationship between procedural functions and behavioral therapies in TS.

The present study is not without limitations. First, the sample size in our study can be considered to be small. At the same time, this sample size corresponds to previous studies that investigated procedural functions in this rare disorder (e.g., Dye et al., 2016; Takács et al., 2017, 2018; Shephard et al., 2019). Second, TS participants in our study are characterized with mild to moderate tic severity and possibly represent the lower end of tic severity dimension as indicated by the YGTSS. In our study, individual differences in procedural memory consolidation did not correlate with tic severity or changes in tic severity over the 1-year offline period in the TS group. Although YGTSS is a well-established clinical measure of tic severity, it would be beneficial to employ additional measures of tic severity in future studies. One potential candidate is the Modified Rush Videotape Rating Scale (Goetz et al., 1999), which is an objective clinical measure of present tic severity. Vocal and motor tics are rated based on their frequency, severity, and distribution. Kleimaker et al. (2020) focused on stimulus-response associations in TS adults and employed both the YGTSS and the Modified Rush Videotape Rating Scale. They revealed stronger stimulus-response binding in TS adults, which resulted in difficulties in unbinding and rebinding established associations. Individual differences in stimulus-response binding did not show correlation with YGTSS scores, however, they correlated with motor tic frequency, that is, with the number of motor tics per minute as measured by the Modified Rush Videotape Rating Scale. Hence, it is possible that finer markers of tics might be related to procedural memory consolidation as well. Non-etheless, our results are limited to a specific TS population and further studies should investigate whether procedural memory consolidation is intact in children with severe tic symptoms as well.

Another intriguing future direction could be a detailed characterization of the temporal dynamics of consolidation. The present study does not provide information about exactly when the consolidation of the acquired information happens. Quentin et al. (2021) showed that learning of probability-based regularities occurs during the online periods, and no further gain is acquired during the offline periods. In contrast, memories of serial-order regularities are formed during the offline periods of the ASRT task. Further studies should explore in detail the temporal dynamics of learning probability-based and serial-order regularities in TS.

## Conclusion

The goal of the present study was to investigate the consolidation of procedural memory in TS. The representation of probability-based regularities remained stable over both a short-term (5-hour) and long-term (1-year) offline period in children with TS and typically developing controls. Both the TS and the control group successfully retained knowledge of probability-based information after the offline periods with comparable memory performance between the groups. In conclusion, procedural memory consolidation seems to be intact in TS even after a 1-year offline period that did not include additional practice. This finding suggests that individuals with TS might be more proficient in skill acquisition as they are able to successfully maintain and retain the learned skills, even over a long period of time.

## Data Availability Statement

The raw data supporting the conclusions of this article can be found in the Supplementary Material.

## Ethics Statement

The studies involving human participants were reviewed and approved by Research Ethics Committee of Eötvös Loránd University. Written informed consent to participate in this study was provided by the participants’ legal guardian/next of kin.

## Author Contributions

ET-F designed the study, collected the data and supervised data acquisition, analyzed data, contributed to the interpretation of the results, and wrote and revised the manuscript. ZT designed the study, recruited the participants in the clinical group, conducted the clinical interviews, and revised the manuscript. ÁT designed the study, contributed to the interpretation of the results, and wrote and revised the manuscript. KJ designed the study, created the scripts running the experiments, contributed to the interpretation of the results, and wrote and revised the manuscript. DN designed the study, contributed to the interpretation of the results, and wrote and revised the manuscript. All authors read and approved the final version of the manuscript.

## Conflict of Interest

The authors declare that the research was conducted in the absence of any commercial or financial relationships that could be construed as a potential conflict of interest.

## Publisher’s Note

All claims expressed in this article are solely those of the authors and do not necessarily represent those of their affiliated organizations, or those of the publisher, the editors and the reviewers. Any product that may be evaluated in this article, or claim that may be made by its manufacturer, is not guaranteed or endorsed by the publisher.
