# Supplementary material for: Access to Procedural Memories After One Year: Evidence for Robust Memory Consolidation in Tourette Syndrome
Source: Front Hum Neurosci. 2021 Aug 12;15:715254. doi: 10.3389/fnhum.2021.715254 (PMC8407083; doi:10.3389/fnhum.2021.715254)
Supplement: Supplementary file 2 [file Data_Sheet_2.docx]

**Supplementary Materials**

**Analyses of the short- and long-term consolidation of serial-order knowledge**

Analyses on performance in the Learning Phase have shown that the participants did not acquire the serial-order information (see Prerequisite of memory consolidation section in the Manuscript). Hence, the prerequisite of memory consolidation did not fulfill which calls into question the applicability of retention analyses concerning sequence learning. Nevertheless, in sake of completeness, we report the analyses on short- and long-term retention of serial-order knowledge here.

**Short-term (five-hour) consolidation of serial-order knowledge**

To test the five-hour retention of serial-order knowledge, we run a mixed-design ANOVA on RT with GROUP (TS vs TD) as between-subjects factor and ORDER (pattern vs random high) and EPOCH (4 vs 5) as within-subject factors. Overall, irrespective of epochs and group, participants were faster on pattern high (*M* = 386.37 ms) than on random high trials (*M* = 415.70 ms) (main effect of ORDER, *F*(1, 36) = 5.58, *p* < .02, *η^2^_p_ = .*13). The ANOVA revealed retained serial-order memory after the five-hour delay (non-significant ORDER × EPOCH interaction, *F*(1, 36) = 2.20, *p* = .15, *BF_01_* = 2.06), memory scores were similar in the 4^th^ (*M* = 22.22 ms) and 5^th^ (*M* = 36.43 ms) epochs. The TS and TD groups showed comparable memory performance (non-significant GROUP × ORDER × EPOCH interaction, *F*(1, 36) = 0.52, *p* = .48, independent samples t-tests were conducted on the short-term offline change score, *BF_01_* = 2.59, short-term offline change scores: *M_TS_* = 21.11 ms, *M_TD_* = 7.32 ms). Other main effects or interactions were also not significant (all *p*s > .070).

**Long-term (one-year) consolidation of serial-order knowledge**

To test one-year retention of sequential knowledge, we conducted a mixed design ANOVA on RT with GROUP (TS vs TD) as between-subjects factor and ORDER (pattern vs random high) and EPOCH (6 vs 7) as within-subject factors. Irrespective of group and epochs, participants showed faster RTs on pattern high (*M* = 370.03 ms) than on random high trials (*M* = 412.08 ms) (main effect of ORDER, *F*(1, 36) = 5.34, *p* = .03, *η^2^_p_ = .*13). The ANOVA revealed that, over groups, the memory scores did not change in the one-year-long offline period (non-significant ORDER × EPOCH interaction, *F*(1, 36) = 1.90, *p* = .18, *BF_01_* = 2.41), with similar memory scores in the 6th (*M* = 58.65 ms) and in the 7th (*M* = 25.45 ms) epochs. The TS and TD groups did not differ in retention (non-significant GROUP × ORDER × EPOCH interaction, *F*(1, 36) = 1.20, *p* = .28, independent samples t-tests were conducted on the long-term offline change score *BF_01_* = 1.98, long-term offline change scores: *M_TS_* = -59.61 ms, *M_TD_* = -6.79 ms). Other main effects or interactions were not significant (all *p*s > .074).
